# A bioinformatics pipeline for identifying clinically relevant immune and adhesion biomarkers in thyroid risk stratification

**DOI:** 10.3389/fmolb.2026.1814666

**Published:** 2026-06-23

**Authors:** Larissa Teodoro Rabi, Karina Colombera Peres, Elisangela De Souza Teixeira, Alfio José Tincani, Natassia Elena Bufalo, Laura Sterian Ward

**Affiliations:** 1 Laboratory of Cancer Molecular Genetics, Faculty of Medical Sciences, State University of Campinas (UNICAMP), Campinas, Brazil; 2 Department of Biomedicine, Nossa Senhora do Patrocínio University Center (CEUNSP), Itu, Brazil; 3 Department of Medicine, Max Planck University Center, Campinas/São Paulo, Brazil; 4 Department of Surgery, Faculty of Medical Sciences, State University of Campinas (UNICAMP), Campinas, Brazil; 5 Department of Medicine, São Leopoldo Mandic and Research Center, Campinas/São Paulo, Brazil

**Keywords:** bioinformatics, cell adhesion molecules, follicular thyroid lesions, *in silico* analysis, molecular biomarkers, single nucleotide polymorphisms, thyroid cancer

## Abstract

The substantial biological heterogeneity of thyroid cancer, particularly within follicular-patterned lesions, underscores the need for improved molecular tools for risk stratification. Genetic variability in the pathways regulating cell adhesion, immune interactions, and extracellular matrix remodeling may influence tumor behavior and the complexity of diagnosis. In this study, we conducted integrated *in silico* and genetic analyses to evaluate the role of polymorphisms in genes encoding immunoglobulin superfamily adhesion molecules, integrins, junctional proteins, matrix metalloproteinases, and extracellular matrix-associated proteins. Using multiple bioinformatics platforms, we screened 407,812 polymorphisms across 22 candidate genes and prioritized 133 variants with high predicted functional impact. Eight selected single-nucleotide polymorphisms were genotyped in a cohort of 648 individuals, including patients with benign thyroid nodules (n = 152), malignant thyroid nodules (n = 171), and healthy controls (n = 325). Clinical validation revealed that *MADCAM1* rs3745925 significantly distinguished follicular adenoma from controls (p = 0.017, OR: 3.15; 95% CI: 1.63–6.05), goiter (p = 0.019, OR: 3.18; 95% CI: 1.49–6.85), and papillary thyroid carcinoma (p = 0.009, OR: 3.49; 95% CI: 1.73–7.09). This association remained robust after Bonferroni correction, underscoring its potential as a priority candidate. Additionally, *ITGAM* rs1143683, *ITGAL* rs2230433, and *ICAM1* rs5498 were associated with tumor multifocality (p = 0.0428, p = 0.0008, and p < 0.0001, respectively). These findings demonstrate the feasibility of integrating bioinformatics-driven variant prioritization with clinical validation methods. Among the evaluated polymorphisms, *MADCAM1* rs3745925 emerged as a promising auxiliary biomarker warranting further evaluation for the characterization of follicular-patterned thyroid lesions.

## 1 Introduction

Thyroid cancer (TC) is the most common endocrine malignancy worldwide, with an increasing incidence (Paz-Ibarra et al., 2024; Forma et al., 2025). Although the overall prognosis of differentiated thyroid cancer (DTC) is favorable, a significant subset of patients develops an aggressive disease characterized by local invasion, metastasis, and therapeutic resistance (Forma et al., 2025; Houten et al., 2023). This clinical heterogeneity underscores the need for better molecular stratification tools to distinguish indolent tumors from aggressive ones (Forma et al., 2025).

Genetic susceptibility significantly contributes to interindividual variability in both the risk and clinical outcomes of the disease (Tiucă et al., 2023). Single nucleotide polymorphisms (SNPs) are the most common form of human genetic variation (Saenko and Rogounovitch, 2018). Coding-region SNPs can alter protein structure and function, whereas non-coding variants may influence gene expression and RNA processing (Cargill et al., 1999). Increasing evidence suggests that polymorphisms in genes governing cell adhesion, migration, and extracellular matrix (ECM) remodeling can modulate tumor aggressiveness in DTC, making functionally relevant SNPs promising candidates for use as diagnostic and prognostic biomarkers (Saenko and Rogounovitch, 2018).

Thyroid carcinogenesis and progression are driven by dysregulated cellular processes, including adhesion, migration, and invasion, which depend on complex interactions with the tumor microenvironment (Andriescu et al., 2018; Rabi et al., 2023; Zhao et al., 2019). Cell adhesion molecules (CAMs) are central to these interactions. This heterogeneous group of transmembrane glycoproteins mediates cell-cell and cell-extracellular matrix interactions, governing tissue architecture, signaling, and cell behavior (Ley et al., 2007). Altered expression or function of these proteins can disrupt tissue organization and facilitate dissemination (Andriescu et al., 2018; Rabi et al., 2023; Smart et al., 2021; Chriqui et al., 2025; Burčík et al., 2025).

Within the CAM superfamily, immunoglobulin superfamily CAMs (IgSF-CAMs), such as ICAMs, VCAMs, PECAM-1, MAdCAM-1, and JAMs, are key regulators of leukocyte trafficking, endothelial permeability, and inflammation (Ley et al., 2007; Guerra-Espinosa et al., 2024; Singh et al., 2023). Dysregulated IgSF-CAM expression has been implicated in multiple steps of cancer progression, including invasion, metastasis, and immune evasion (von Lersner et al., 2019; Wai Wong et al., 2012). In thyroid cancer, aberrant adhesion molecule expression is linked to the loss of epithelial polarity, architectural disorganization, and invasive phenotypes, highlighting their potential as biomarkers (Rabi et al., 2023; Ma et al., 2024; Wan et al., 2022; Man et al., 2025; Kim et al., 2023; Yu et al., 2023).

Integrins constitute another major family of adhesion receptors that are critical to tumorigenesis. These heterodimeric transmembrane proteins, composed of α and β subunits, mediate bidirectional signaling between the ECM and cytoskeleton, regulating adhesion, migration, proliferation, and survival (Ley et al., 2007; Bergonzini et al., 2022; Mezu-Ndubuisi and Maheshwari, 2021; Hamidi and Ivaska, 2018). The β2 integrin subfamily (*ITGB2*), associated with α subunits such as *ITGAL, ITGAM*, and *ITGAD*, is primarily leukocyte-specific and mediates interactions with IgSF-CAMs in inflammatory and tumor microenvironments (Guerra-Espinosa et al., 2024). Similarly, *ITGA4* contributes to lymphocyte homing and endothelial adhesion, whereas ITGA10 is involved in collagen binding and tumor-stroma interactions (Li et al., 2025). Altered integrin expression and signaling promote epithelial-mesenchymal transition, motility, and invasion in thyroid tumors (Yuan et al., 2023).

ECM remodeling, a hallmark of tumor progression, facilitates invasion, angiogenesis, and metastasis. Matrix metalloproteinases (MMPs), particularly MMP-9, degrade ECM components and are associated with aggressive features of thyroid cancer, including extrathyroidal extension and lymph node metastasis (Yuan et al., 2023; Winkler et al., 2020). MMP activity is tightly regulated by tissue inhibitors of metalloproteinases (TIMPs), and an MMP–TIMP imbalance creates a permissive microenvironment for cancer progression. Other ECM-associated proteins, such as thrombospondins, further modulate cell adhesion, angiogenesis, and tumor–microenvironmental crosstalk (Niland et al., 2022; Brassart-Pasco et al., 2020).

Given their established roles in tumor dissemination and accessibility for molecular profiling, genes encoding adhesion molecules, integrins, and extracellular matrix components are promising targets for biomarker discovery. Therefore, this study aimed to systematically evaluate the functional impact of polymorphisms in these gene families using an integrated *in silico* framework complemented by validation in clinical thyroid nodule specimens.

## 2 Materials and methods

### 2.1 In silico analysis

This study comprised an *in silico* analysis of genetic polymorphisms and their impact on DNA and/or protein structure using multiple bioinformatics tools for the functional, structural, and stability characterization of the evaluated alterations, as previously described by our group (Rabi et al., 2024; Peres et al., 2021; Nascimento et al., 2023; Viana et al., 2025; Bufalo et al., 2021; Martins et al., 2021).

**FIGURE 1:**
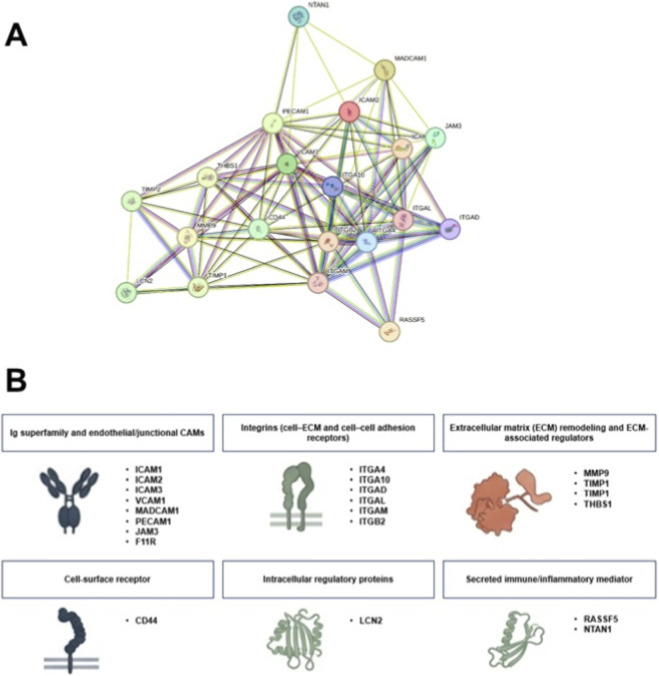
Protein–protein interaction (PPI) network and functional classification of adhesion and ECM-related proteins. **(A)** PPI network highlighting interactions among CAMs, integrins, ECM remodeling regulators, and associated proteins (String Tool). **(B)** Functional grouping of genes evaluated in this study.

Twenty-two genes were selected for analysis (Figure 1), including immunoglobulin superfamily cell adhesion molecules (*ICAM1, ICAM2, ICAM3, MADCAM1, PECAM1, VCAM1, JAM3, and F11R*), integrins (*ITGA4, ITGA10, ITGAD, ITGAL, ITGAM, and ITGB2*), extracellular matrix remodeling proteins (*MMP9, TIMP1, TIMP2, and THBS1*), cell surface receptors (*CD44*), intercellular regulatory proteins (*LCN2*), and secreted immune/inflammatory mediators (*NTAN1 and RASSF5*).

SNPs data were obtained from the Single Nucleotide Polymorphism Database (dbSNP) (Phan et al., 2025) of the National Center for Biotechnology Information (NCBI). For each gene, information was collected on SNP identifier (rsID), chromosomal position, reference, and polymorphic alleles of the gene. Structural and functional information of the proteins encoded by the genes of interest was obtained from the Universal Protein Resource Database (UniProt) (UniProt Consortium, 2025), including primary amino acid sequence (FASTA), functional domains, subcellular localization, biological function, and three-dimensional structures modeled on the AlphaFold (Fleming et al., 2025) platform.

### 2.2 Analysis of SNPs in non-coding regions

SNPs in non-coding regions (Table 1), including promoters, enhancers, 5′and 3′UTRs, and intronic sites, were analyzed using PredictSNP 2.0 (Bendl et al., 2016), which integrates multiple predictive algorithms: Combined Annotation Dependent Depletion (CADD) (Kircher et al., 2014), deep learning annotation of genetic variants (DANN) (Quang et al., 2015), functional analysis through hidden Markov models-multiple kernel learning (FATHMM-MKL) (Shihab et al., 2015), FunSeq2 (Fu et al., 2014), and genome-wide annotation of variants (GWAVA) (Ritchie et al., 2014).

**TABLE 1:** Computational tools and databases employed for *in silico* functional prediction and structural analysis of genetic variants.

ID	Access link	Purpose/Prediction	Methodology
dbSNP	https://www.ncbi.nlm.nih.gov/snp/	Public archive for genetic variation and SNPs	Curated database integrating submissions from researchers and large-scale sequencing projects
UniProt	https://www.uniprot.org/	Protein sequence and functional information database	Curated database combining Swiss-Prot and TrEMBL
AlphaFold	https://alphafold.ebi.ac.uk/	Provides highly accurate protein structure predictions	Deep learning system using NN
PredictSNP2.0	https://loschmidt.chemi.muni.cz/predictsnp2/	Consensus classifier for predicting pathogenicity of variants in non-coding and coding regions	Meta-predictor
CADD		Scores deleteriousness of SNPs	Integrates multiple annotations using SVM
DANN		Pathogenic effects of SNPs in non-coding regions	Deep neural network
FATHMM-MLK		Functional consequences	Combined hidden Markov models with machine learning
FunSeq2		Prioritizes functional variants in non-coding regions	Integrates genomic context and functional annotations using weighted scoring system
GWAVA		Predicts impact of non-coding genetic variants	Random forest classifier
PredictSNP1.0	https://loschmidt.chemi.muni.cz/predictsnp1/	Consensus classifier for missense variant pathogenicity prediction	Meta-predictor
MAPP		Impact of amino acid substitutions on protein function	Physicochemical variation analysis in multiple sequence alignments
PhD-SNP		Disease-associated missense mutations	SVM
PolyPhen-1		Functional effects of amino acid substitutions	Sequence-based and structure-based features with naive bayes classifier
PolyPhen-2		Impact of amino acid substitutions on protein structure and function	Machine learning
SIFT		Functional effects of amino acid substitutions	Sequence homology-based approach using normalized probabilities from sequence alignments
PANTHER		Functional effects of amino acid substitutions	Phylogenetic-based approach combined with hidden markov models
SNAP		Functional effects of amino acid substitutions	NN trained on protein sequence and evolutionary information
DynaMut2.0	http://biosig.unimelb.edu.au/dynamut2/	Impact of amino acid substitutions on protein stability and dynamics	Graph-based signatures integrating structural flexibility
MuPRO	http://mupro.proteomics.ics.uci.edu/	Impact of amino acid substitutions on protein stability	SVM
iStable	http://predictor.nchu.edu.tw/iStable/	Impact of amino acid substitutions on protein stability	Meta-predictor
NetSurf	https://services.healthtech.dtu.dk/services/NetSurfP-3.0/	Surface accessibility and secondary structure of proteins	Deep NN
ConSurf	https://consurf.tau.ac.il/	Evolutionary conservation analysis	Phylogenetic analysis of homologous sequences
NetPhos3.1	https://services.healthtech.dtu.dk/services/NetPhos-3.1/	Serine, threonine, and tyrosine phosphorylation sites	NN trained on experimentally verified phosphorylation sites
Musite	http://musite.net/	PTM sites including phosphorylation	SVM with disorder and evolutionary information
ProP1.0	https://services.healthtech.dtu.dk/services/ProP-1.0/	Arginine and lysine propeptide cleavage sites	NN trained on cleavage site sequences
NetNGlyc1.0	https://services.healthtech.dtu.dk/services/NetNGlyc-1.0/	Predicts N-glycosylation sites in proteins	Artificial NN trained on experimentally verified glycosylation sites
NetOGlyc1.0	https://services.healthtech.dtu.dk/services/NetOGlyc-4.0/	O-glycosylation sites in mammalian proteins	NN using sequence context and surface accessibility
GPS6.0	http://gps.biocuckoo.cn/	Kinase-specific phosphorylation sites	Group-based scoring algorithm with kinase-specific motifs and evolutionary information
DeepLoc2.1	https://services.healthtech.dtu.dk/services/DeepLoc-2.1/	Subcellular localization of eukaryotic proteins	DL using attention mechanisms on protein sequences
DeepTMHMM	https://dtu.biolib.com/DeepTMHMM	Transmembrane topology and signal peptides	DL combining convolutional NN and hidden markov models

SNPs, Single Nucleotide Polymorphisms; NN, Neural Network; SVM, Support Vector Machine; PTM, Post Translational Modifications and DL, Deep Learning.

### 2.3 Analysis of missense SNPs

Missense SNPs (Table 1), which result in amino acid substitutions in the protein sequence, were evaluated using the PredictSNP 2.0 platform (Bendl et al., 2016) for DNA impacts and subjected to pathogenicity and functional impact predictive analyses using a robust set of platforms. The PredictSNP 1.0 platform (Bendl et al., 2014) was used to assess the deleterious potential of missense variants by integrating six predictive tools based on different algorithmic principles: MAPP (Multivariate Analysis of Protein Polymorphism) (Stone and Sidow, 2005); PhD-SNP (Predictor of human Deleterious Single Nucleotide Polymorphisms) (Capriotti et al., 2006); PolyPhen-1 (Ramensky et al., 2002); PolyPhen-2 (Adzhubei et al., 2010); SIFT (Sorting Intolerant From Tolerant) (Ng and Henikoff, 2003); PANTHER (Brunham et al., 2005) and SNAP (Screening for Non-Acceptable Polymorphisms) (Bromberg and Rost, 2007).

### 2.4 Protein stability analysis

DynaMut 2.0 (Rodrigues et al., 2021), MuPRO (Protein Stability Prediction upon Mutation) (Cheng et al., 2006) and iStable (Chen et al., 2013) platforms were used to classify mutations as stabilizing or destabilizing with associated confidence values (Table 1).

### 2.5 Analysis of structural and functional protein characteristics

Evolutionary conservation analysis was performed using the NetSurf (Høie et al., 2022) and ConSurf (Glaser et al., 2003) servers. In addition, phosphorylation sites on serine, threonine, and tyrosine were predicted using NetPhos 3.1 (Blom et al., 1999; Blom et al., 2004) and Musite (Wang et al., 2020; Wang et al., 2019; Wang et al., 2017). Propeptide Cleavage Sites 1.0 (Duckert et al., 2004) was used to identify the proteolytic cleavage sites. N-glycosylation sites were predicted using NetNGlyc 1.0 (Gupta and Brunak, 2002), and O-glycosylation sites on serine and threonine were predicted using NetOGlyc 4.0.0.13 (Steentoft et al., 2013). GPS 6.0 (Group-based Prediction System) (Chen et al., 2023) predicted various post-translational modifications, including acetylation, methylation, ubiquitination, and sumoylation, and identified variants that potentially alter the modification profiles. Subcellular localization was predicted using DeepLoc 2.1 (Nielsen, 2025), and membrane topology and transmembrane domains were assessed using DeepTMHMM (Hallgren et al., 2022) (Table 1).

### 2.6 Validation in thyroid nodules

This study was conducted in accordance with the ethical principles for human research and was approved by the Research Ethics Committee of the Faculty of Medical Sciences, University of Campinas (CEP-FCM/UNICAMP) under protocol CAAE 70994623.9.0000.5404. Biological material was obtained from the Biorepository of the Laboratory of Cancer Molecular Genetics (GEMOCA) at FCM/UNICAMP, comprising previously extracted genomic DNA samples from peripheral blood.

Eight polymorphisms were selected for validation: rs1143676 (ITGA4); rs2230433 (ITGAL), rs1143683 (ITGAM), rs1799969 (ICAM1), rs5498 (ICAM1), rs3745925 (MADCAM1), rs17576 (MMP9) and rs17577 (MMP9). The selection of these specific variants was based on three main criteria: (i) a Minor Allele Frequency (MAF) > 0.1 in the Brazilian population, verified through the Archive of Brazilian Online Mutations (ABRAOM) repository, to ensure adequate statistical power for clinical validation in our specific cohort; (ii) existing scientific literature supporting their functional roles in related pathways; and (iii) deleterious consensus scores across our bioinformatics pipeline.

### 2.7 Study population

**FIGURE 2:**
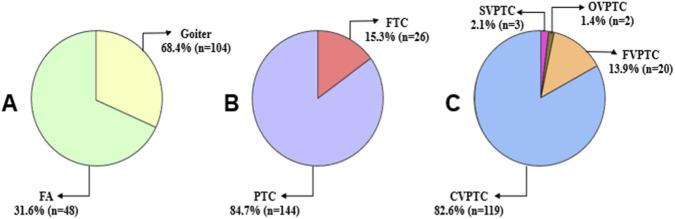
Distribution of histopathological diagnoses in the benign and malignant groups. **(A)** Benign lesions: goiter (n = 104) and FA (n = 48). **(B)** Malignant tumors: PTC (n = 144) and FTC (n = 26). **(C)** PTC variants: CVPTC (n = 119), FVPTC (n = 20), OVPTC (n = 2), and SVPTC (n = 3).

The study population comprised three groups: patients with benign nodules (n = 152), patients with malignant tumors (n = 171), and healthy controls (n = 325). The distribution of histopathological diagnoses is shown in Figure 2. The benign nodule group had a mean age of 48.3 ± 14.1 years and mean nodule size of 2.13 ± 1.19 cm. This group was predominantly female (89.5%; n = 136). The diagnoses consisted of goiter (68.4%; n = 104) and follicular adenoma (FA; 31.6%; n = 48) (Figure 2A). Patients in the malignant group had a mean age of 42.0 ± 12.6 years and a mean tumor size of 1.77 ± 1.05 cm, with 81.2% (n = 138) of patients being women. The histopathological features included multifocality (31.5%; n = 52), associated chronic lymphocytic thyroiditis (40.4%; n = 46), invasion (37.1%; n = 56), and metastasis at diagnosis (25.4%; n = 30). The majority of cases were papillary thyroid carcinoma (PTC; 84.7%; n = 144), with the remainder being follicular thyroid carcinoma (FTC; 15.3%; n = 26) (Figure 2B). Among the PTC cases, the classic variant was the most frequent (CVPTC; 82.9%; n = 119), followed by follicular (FVPTC; 13.9%; n = 20), sclerosing (SVPTC; 2.1%; n = 3), and oxyphilic variants (OVPTC; 1.4%; n = 2) (Figure 2C). The control group (n = 325) consisted of age- and sex-matched individuals with no history of thyroid disease, with a mean age of 42.8 ± 11.9 years, and 84.9% (n = 276) of the participants were women.

### 2.8 Genotyping methodology

Genotype determination was performed using TaqMan® SNP Genotyping Assays (Applied Biosystems™) on a 7300 Real-Time PCR System (Applied Biosystems™, Foster City, United States). Commercially available pre-validated assays (TaqMan® Pre-Designed SNP Genotyping Assays, Applied Biosystems™) were used to ensure specificity and reproducibility (*ITGA4*, rs1143676, C___1276216_1_; *ITGAL*, rs2230433, C__11789692_10; *ITGAM*, rs1143683, C__11388882_20; *ICAM1*, rs1799969, C___8726331_10 and rs5498, C___8726337_40; *MADCAM1*, rs3745925, C__27472568_10 and *MMP9*, rs17576, C__11655953_10 and rs17577, C__11655948_1_).

### 2.9 DNA quantification and quality control

Genomic DNA was quantified by spectrophotometry using an Epoch Microplate Spectrophotometer (BioTek Instruments) and Gen5 software. Concentrations were determined by measuring absorbance at 260 nm, and purity was verified using the A260/A280 and A260/A230 ratios within the acceptable ranges (1.8–2.0 and >1.8, respectively). The samples were then diluted to a standardized concentration of 10 ng/μL with ultrapure water and quantified. Only samples with confirmed concentrations between 5 and 15 ng/μL were processed further; those outside this range were adjusted and re-evaluated until they met this criterion.

### 2.10 PCR amplification

Each amplification reaction was performed in a final volume of 7 μL, containing 20 ng of genomic DNA, 3.5 μL TaqMan™ Genotyping Master Mix (2X, final concentration 1X), 0.175 μL TaqMan-specific assay (40X, containing primers and fluorescent probes, final concentration 1X), and 1.325 μL ultrapure water (Milli-Q). Negative controls containing ultrapure water instead of DNA were included in all runs to monitor contamination and to validate reaction specificity. The thermal cycling conditions were as follows: initial polymerase activation at 95 °C for 10 min, followed by 40 cycles of denaturation at 92 °C for 15 s and annealing/extension at 60 °C for 90 s. Allelic discrimination and genotype determination were performed using 7500 Software version 2.3 (Applied Biosystems™, Foster City, United States), following the manufacturer’s recommendations. Individuals were classified into three genotypic categories based on their characteristic fluorescence patterns: homozygous for the reference allele (wild-type), heterozygous, and homozygous for the polymorphic allele.

### 2.11 Statistical analysis

Descriptive statistics (mean, standard deviation, median, and range) were used to summarize the sample profiles. Between-group comparisons for continuous and categorical variables were performed using the Mann-Whitney U test and chi-square or Fisher’s exact test, respectively. A two-sided p-value <0.05 was considered statistically significant for all tests, and all p-values were corrected using the Bonferroni method, when appropriate.

## 3 Results

### 3.1 Bioinformatics analysis

The SNPs registered for twenty-one candidate genes were systematically evaluated in this study. Initial screening identified 407,812 polymorphisms reported in the dbSNP platform, of which 133 SNPs and 207 nucleotide substitutions were selected as potential targets for future validation in TC based on predefined selection criteria. Comprehensive details of the screening process and the selected variants are presented in Table 2. The most functionally relevant variants are discussed herein, with an emphasis on those that demonstrate the highest pathogenic potential.

**TABLE 2:** Summary of *in silico* analysis: evaluated and potential target SNPs identified across twenty-two candidate genes.

Gene	Total reported in dbSNP	MAF>0.1	After duplicate and merged removal SNPs (N alt)	Potential targets		Missense variants MAF>0.1	
				N SNPs (N alt)	rsID	N SNPs (N alt)	rsID
*ICAM1*	8819	104	21 (41)	1 (2)	rs923366	1 (2)	rs5498
*ICAM2*	9040	61	19 (48)	3 (6)	rs4968683, rs4968605, rs16947352	0	-
*ICAM3*	4809	62	25 (42)	1 (1)	rs7250300	2 (3)	rs2304237 rs7258015
*VCAM1*	9397	71	21 (36)	2 (4)	rs3917010, rs2392221	0	-
*MADCAM1*	7109	59	33 (47)	1 (3)	rs758503	1 (2)	rs3745925
*PECAM1*	28,421	115	38 (80)	3 (6)	rs9902260, rs1470453, rs12941698	0	-
*JAM3*	44,370	656	183 (310)	20 (32)	rs621698, rs659196, rs610382, rs7936421, rs595102, rs11223686, rs1143809, rs470966, rs12287552, rs470846, rs470417, rs647837, rs470456, rs1143811, rs637517, rs7101690, rs11223703, rs11223709, rs2276266, rs610829	0	-
*F11R*	12,276	182	54 (89)	8 (14)	rs28365979, rs28365980, rs28365978, rs2481085, rs790055, rs11807930, rs2988726, rs2481084	0	-
*ITGA4*	39,162	530	175 (308)	15 (21)	rs155102, rs17290693, rs3770138, rs2305586, rs62191453, rs3770109, rs113276800, rs4667308, rs16867433, rs3770108, rs2305588, rs3770116, rs6748657, rs16867438, rs10490691	2 (5)	rs1143676 rs7562325
*ITGA10*	10,920	36	13 (10)	0	-	0	-
*ITGAD*	16,534	103	30 (62)	1 (3)	rs8050500	0	-
*ITGAL*	24,144	254	64 (127)	7 (14)	rs11150589, rs12716977, rs11574938, rs12598978, rs11574941, rs1557672, rs4341759	1 (1)	rs2230433
*ITGAM*	33,448	366	137 (228)	10 (14)	rs35314490, rs9940397, rs4889647, rs9939679, rs140615691, rs9928749, rs7198598, rs11865746, rs11864503, rs9933520	2 (2)	rs1143678 rs1143683
*ITGB2*	22,629	398	140 (243)	6 (7)	rs2280965, rs2838727, rs59498405, rs2072703, rs2072702, rs7281781	0	-
*MMP9*	5803	97	22 (35)	6 (8)	rs3918262, rs9509, rs2274755, rs20544, rs3918240, rs3918253, rs17576, rs2250889	3 (8)	rs17576 rs17577 rs2250889
*TIMP1*	2381	20	7 (13)	1 (2)	rs6609534	1 (2)	rs4898
*TIMP2*	33,003	489	259 (338)	6 (9)	rs7222198, rs4789936, rs12452379, rs8179096, rs72849591, rs9912418	0	-
*THBS1*	9653	79	20 (35)	3 (3)	rs1478605, rs2664139, rs7170682	2 (5)	rs2228261 rs2292305
*CD44*	39,602	515	156 (276)	23 (37)	rs10836343, rs12365962, rs11033008, rs11033010, rs353624, rs353620, rs353644, rs353629, rs353628, rs3794110, rs11033022, rs10836340, rs4756199, rs12362973, rs353558, rs2553808, rs1981154, rs353612, rs353627, rs3794109, rs2295756, rs10734436, rs74938975	2 (6)	rs1071695 rs9666607
*LCN2*	3296	8	6 (11)	0	-	0	-
*RASSF5*	32,526	319	101 (172)	14 (18)	rs12124627, rs4845112, rs67223538, rs72755019, rs7530746, rs11119043, rs11119083, rs11589, rs71633591, rs871782, rs3813976, rs4844540, rs6660116, rs7527917	1 (2)	rs4845112
*NTAN1*	10,470	123	37 (70)	3 (3)	rs1135999, rs11075253, rs2280017	2 (5)	rs1135999 rs1136001
TOTAL	407,812	4647	1561 (2621)	133 (207)		20 (43)	

dbSNP, SNP databank NCBI; MAF, Minor Allele Frequency; N Alt, Nucleotide alteration.

### 3.2 Immunoglobulin superfamily cell adhesion molecules (ICAM1, ICAM2, ICAM3, MADCAM1, PECAM1, VCAM1, JAM3 and F11R)

Analysis of *ICAM1, ICAM2*, and *ICAM3* revealed recurrent but gene-specific patterns of predicted functional impact. In ICAM1, rs923366 showed moderate deleterious potential (33.3%, n = 2; FunSeq and GWAVA) and predicted regulatory effects. The missense variant rs5498 (A>G at position 10285007; K469E) showed limited consensus pathogenicity (PredictSNP2.0, 16.7%, n = 1; GWAVA, 100% neutral by PredictSNP1.0, Table 3) but was localized to an exposed (NetSurf and ConSurf), proteolytic cleavage site (ProP Score = 0.098), and harbored post-translational modification sites including ubiquitination (0.294), sumoylation (0.045), N6-acetyllysine (0.205), methyllysine (0.081), and hydroxylysine (0.031) (Musite). In addition, reduced protein stability (MuPRO ΔΔG = −0.6109; iStable = 0.5103) and the addition of an interaction with GLY405, which is absent in the wild-type structure, promote charge alterations that potentially disrupt molecular contacts (HOPE).

**TABLE 3:** Pathogenicity prediction and protein stability analysis of missense variants using PredictSNP1.0 and MuPRO.

Gene	rsID	AA change	PredictSNP	MAPP	PhD-SNP	PolyPhen-1	PolyPhen-2	SIFT	SNAP	nsSNPAnalyzer	Panther	MuPRO
ICAM1	rs5498	K469E	N	N	N	N	N	N	N	U	N	−0,61
ICAM3	rs7258015	R115G	N	N	N	N	N	N	N	U	N	−1,21
ICAM3	rs2304237	D143G	N	N	N	N	N	N	N	U	N	−1,17
ICAM3	rs2304237	D143A	N	D	N	N	N	D	N	U	N	−0,70
MADCAM1	rs3745925	P300H	N	N	N	D	D	N	D	U	N	−0,72
MADCAM1	rs3745925	P300R	D	D	N	D	D	N	D	U	N	−0,54
ITGA4	rs1143676	R878Q	N	N	N	N	N	N	N	U	N	−1.35
ITGA4	rs1143676	R878L	N	N	D	N	N	D	N	U	N	0.01
ITGA4	rs7562325	H961Q	N	N	N	N	N	N	N	U	N	−0.90
ITGAL	rs2230433	R791T	N	N	N	N	N	N	N	U	N	−0.51
ITGAM	rs1143678	P1147S	N	N	N	N	N	N	N	U	U	−0.13
ITGAM	rs1143683	A858V	N	D	N	N	N	N	N	U	U	−0.57
MMP9	rs17576	Q279P	D	U	D	D	D	N	N	U	U	−1.94
MMP9	rs17576	Q279R	N	U	N	N	N	N	D	U	U	−1.62
MMP9	rs17576	Q279L	N	N	N	N	N	N	D	U	U	−1.03
MMP9	rs17577	R668Q	N	N	N	N	N	N	N	U	N	−0.53
MMP9	rs17577	R668P	N	D	D	N	N	N	D	U	N	−0.73
MMP9	rs2250889	R574Q	N	N	N	N	N	D	N	U	N	−0.52
MMP9	rs2250889	R574P	N	N	N	N	N	N	N	U	N	−0.88
MMP9	rs2250889	R574L	N	N	N	N	N	N	N	U	N	0.11
TIMP1	rs4898	F124L	N	D	D	N	N	N	N	U	U	−0.49
THBS1	rs2228261	N470K	N	N	D	N	N	D	D	U	N	−0.75
THBS1	rs2292305	T523P	N	N	N	N	N	N	N	U	N	−1.13
THBS1	rs2292305	T523A	N	N	N	N	N	N	N	U	N	−0.76
CD44	rs1071695	H85Q	N	D	D	N	N	N	D	U	N	−0.79
CD44	rs9666607	K417T	N	N	N	N	N	N	N	U	N	−0.32
CD44	rs9666607	K417R	N	N	N	N	N	N	N	U	N	−0.21
CD44	rs9666607	K417I	D	U	D	D	N	N	D	U	N	0.13
RASSF5	rs4845112	R277W	D	D	D	D	D	D	D	U	D	−0.67
NTAN1	rs1135999	S287A	N	D	N	N	N	N	N	U	N	−0.87
NTAN1	rs1135999	S287P	N	N	N	D	N	N	N	U	N	−1.00
NTAN1	rs1136001	H283Y	N	N	N	N	N	N	N	U	N	−0.84
NTAN1	rs1136001	H283D	N	N	N	N	N	N	N	U	N	−1.08
NTAN1	rs1136001	H283A	N	N	N	N	N	N	N	U	N	−1.18

AA, amino acid; N, neutral; D, Deleterious and U, unknown.

In *ICAM2*, three variants (rs4968683: A>C, A>G, and A>T; rs4968605: A>C and A>T; and rs16947352: C>T, C>A, and C>G) demonstrated a higher deleterious consensus (50%, n = 3, PredictSNP2.0, CADD, and DANN) across the PredictSNP2.0 consensus, suggesting potential structural or regulatory relevance of these variants. Similarly, in *ICAM3*, rs7250300 (C>A) exhibited moderate deleterious potential (50%, n = 3; PredictSNP2.0, CADD and DANN). Structural evaluation of rs2304237 (T>C; D143A or T>G; D143G) revealed that the D143A substitution was classified as 22.2% deleterious (n = 2; MAPP, SIFT) by PredictSNP1.0 (Table 3). Both demonstrated reduced stability (D143A ΔΔG = −0.703 and D143G ΔΔG = −1.171, MuPRO) and loss of residue interactions (ARG115), despite partial neutrality classification (Dynamut2.0). An additional *ICAM3* missense variant, rs7258015 (T>C, R115G), despite a partial neutrality classification (PredictSNP2.0, 16,7% n = 1 GWAVA and 100% neutral PredictSNP1.0, Table 3), further demonstrated destabilization (MuPRO ΔΔG = −1.21; iStable 0.5669) and disruption of multiple residue contacts in an exposed and functionally annotated region (ALA45, TYR89, and ASP143, Dynamut2.0). Position 115 was characterized as an exposed region (NetSurf, ConSurf), cleavage site (ProP 0.035), transmembrane region (DeepLoc 0.9700), and methylarginine modification site (Musite 0.053).

In *MADCAM1*, rs758503 showed moderate deleterious potential (33.3%, n = 2; FunSeq, GWAVA). Missense substitutions at position 300 (rs3745925; C>A,G; P300 H/R) were inconsistently classified across predictors (PredictSNP2.0 100% neutral and 37,5% deleterious n = 3; PolyPhen-1, PolyPhen-2, SNAP in PredictSNP1.0, Table 3) but were uniformly associated with reduced protein stability (MuPRO ΔΔG = −0.7155; iStable 0.5625). Position 300 is located in an exposed region (NetSurf, ConSurf), classified as transmembrane (DeepLoc 0.9670), and harbors a hydroxyproline modification site (Musite 0.153).

In *PECAM1*, rs9902260 (C>A, C>G, C>T) and rs1470453 (T>A) exhibited a high deleterious consensus (66.7%, n = 4; PredictSNP2.0, CADD, FATHMM and FunSeq2), whereas rs12941698 (T>A, T>C) showed a moderate predicted impact (50%, n = 3; PredictSNP2.0, CADD, DANN). *VCAM1* harbored some of the most pathogenic variants identified, particularly rs3917010 (A/G), classified as 100% deleterious, along with rs2392221 (C>A), which showed a high deleterious consensus (66.7%, n = 4; PredictSNP2.0, CADD, FATHMM, and FunSeq2).

All evaluated *JAM3* polymorphisms demonstrated uniformly high pathogenic potential. Variants rs621698 (G>A), rs659196 (T>A, T>C, T>G), rs610382 (T>A, T>C), and rs7936421 (T>C) were consistently classified as ≥83.3% deleterious (n = 5; PredictSNP2.0, CADD, DANN, FATHMM, and FunSeq2), indicating a substantial predicted functional or regulatory effect. Among all the analyzed genes, *F11R* exhibited the highest pathogenic burden. The rs28365979 (C>T) variant was classified as 100% deleterious by PredictSNP2.0, whereas multiple additional substitutions (rs28365980, rs28365978, rs2481085, rs790055, rs11807930, rs2988726, and rs2481084) consistently demonstrated ≥83.3% (n = 5) deleterious consensus, indicating widespread susceptibility to functional disruption across this locus.

### 3.3 Integrins (ITGA4, ITGA10, ITGAD, ITGAL, ITGAM and ITGB2)

Analysis of *ITGA4* revealed 15 non-coding variants (rs155102, rs17290693, rs3770138, rs2305586, rs62191453, rs3770109, rs113276800, rs4667308, rs16867433, rs3770108, rs2305588, rs3770116, rs6748657, rs16867438 and rs10490691) demonstrating a high deleterious consensus, suggesting widespread regulatory susceptibility. Two missense substitutions (rs1143676, R878Q and R878L; and rs7562325, H961Q) showed variable pathogenesis. Structural evaluation revealed that R878Q (100% neutral by PredictSNP1 - Table 3) reduced protein stability (MuPRO ΔΔG = −0.8957) and altered critical residue interactions (Glu930; Dynamut). R878L was considered 22.2% (n = 2, PhdSNP and SIFT, Table 3) deleterious with increased stability (MuPRO ΔΔG = 0.0093). The variant rs7562325 (H961Q; 100% neutral in PredictSNP1.0, Table 3) demonstrated destabilization effects (MuPRO ΔΔG = −0.8434) and disrupted residue contacts essential for integrin-mediated cell adhesion (Val959). In contrast, *ITGA10* showed no variants that met the threshold criteria (≥50% deleterious). *ITGAD* harbored a single non-coding variant (rs8050500, T>A,C,G) with high deleterious potential (83.3%, n = 5, PredictSNP2.0, CADD, DANN, FUNSEQ, and GWAVA), suggesting a regulatory impact on integrin αD expression.

*ITGAL* demonstrated seven non-coding variants (rs11150589, rs12716977, rs11574938, rs12598978, rs11574941, rs1557672, and rs4341759) that were consistently classified as deleterious by multiple prediction platforms. The missense substitution rs2230433 (R791T, 100% neutral by PredictSNP1.0, Table 3) was localized to a functionally critical domain and reduced protein stability (MuPRO ΔΔG = −0.51).

Analysis of *ITGAM* revealed ten non-coding variants (rs35314490, rs9940397, rs4889647, rs9939679, rs140615691, rs9928749, rs7198598, rs11865746, rs11864503 and rs9933520) demonstrating a high deleterious consensus across the PredictSNP2.0 consensus. Two missense substitutions (rs1143678 P1147S, and rs1143683 A878V) were identified with variable pathogenicity predictions (P1147S 100% neutral and A878V 16, deleterious only by MAPP; Table 3) and both were associated with reduced protein stability (MuPRO ΔΔG = −0.13 and ΔΔG = −0.57, respectively). Similarly, *ITGB2* harbored six non-coding variants (rs2280965, rs2838727, rs59498405, rs2072703, rs2072702, and rs7281781) with strong predicted regulatory impacts, indicating a potential disruption of integrin β2 expression and susceptibility to leukocyte adhesion deficiency.

### 3.4 Extracellular matrix remodeling proteins (MMP9, TIMP1, TIMP2 and THBS1)

Analysis of *MMP9* revealed eight non-coding variants (rs3918262, rs9509, rs2274755, rs20544, rs3918240, rs3918253, rs17576 and rs2250889) demonstrating a highly deleterious consensus across PredictSNP2.0. Three missense substitutions (rs17576, rs17577, and rs2250889) were identified, with variable pathogenicity predictions and distinct structural effects. rs17576 exhibited the most severe predicted functional consequences in the present study. The Q279P substitution was classified as deleterious by four PredictSNP1.0 tools (PredictSNP, PhD-SNP, PolyPhen-1, and PolyPhen-2; Table 3) with substantial destabilization (MuPRO ΔΔG = −1.94). Alternative substitutions at this position (MuPRO Q279R: ΔΔG = −1.62; Q279L: ΔΔG = −1.03) were also classified as deleterious by SNAP, indicating the critical structural sensitivity of this residue. In contrast, rs17577 (R668Q) was uniformly classified as neutral with modest destabilization (MuPRO ΔΔG = −0.53), while the alternative R668P substitution showed a deleterious classification by three tools (MAPP, PhD-SNP, SNAP - Table 3) with moderate destabilization (MuPRO ΔΔG = −0.73). The variant rs2250889 exhibited a predominantly neutral classification. Both R574P and R574L were classified as 100% neutral (Table 3) with minimal stability changes (ΔΔG = −0.88 and 0.11, respectively, according to MuPRO), whereas R574Q showed a deleterious classification solely by SIFT (MuPRO ΔΔG = −0.52).

*TIMP1* harbored one non-coding variant (rs6609534) with moderate (50%) deleterious potential and one missense substitution (rs4898, F124L) demonstrating inconsistent classification (100% neutral by PredictSNP2.0 and MAPP and PhDSNP classified as deleterious; Table 3), but a potential impact on metalloproteinase inhibitory function with reduced stability (MuPRO ΔΔG = −0.49). In contrast, TIMP2 exhibited the highest regulatory burden among the analyzed genes, with six non-coding variants (rs7222198, rs4789936, rs12452379, rs8179096, rs72849591, and rs9912418) consistently classified as deleterious, indicating a widespread susceptibility to regulatory disruption.

*THBS1* demonstrated three non-coding variants (rs1478605, rs2664139, and rs7170682) with a strong predicted impact and two missense variants (rs2228261 and rs2292305) with variable classifications. Structural analysis revealed that rs2228261(N470K) localizes to a calcium-binding domain, reduces protein stability (MuPRO ΔΔG = −0,75), and alters key residue interactions involved in extracellular matrix binding (PhDSNP, SIFT, and SNAP classified as deleterious; Table 3). Similarly, rs2292305 showed destabilization (MuPRO T523P ΔΔG = −1,13 and T523A ΔΔG = −0,76), despite a partially neutral classification across certain predictors (100% neutral by PredictSNP1; Table 3).

### 3.5 Cell surface receptors (CD44)

Analysis of CD44 revealed extensive predicted functional impact across 22 non-coding variants (rs10836343, rs12365962, rs11033008, rs11033010, rs353624, rs353620, rs353644, rs353629, rs353628, rs3794110, rs11033022, rs10836340, rs4756199, rs12362973, rs353558, rs2553808, rs1981154, rs353612, rs353627, rs3794109, rs2295756, rs10734436, rs74938975) consistently classified as deleterious across PredictSNP2.0. This widespread regulatory susceptibility suggests multiple potential disruption sites that affect CD44 expression and alternative splicing. Two missense substitutions (rs1071695 H85Q and rs9666607 K417T, K417R, and K417I) were identified with variable pathogenicity. rs1071695 (H85Q) was classified as deleterious by three PredictSNP1.0 tools (MAPP, PhD-SNP, and SIFT) with moderate destabilization (MuPRO ΔΔG = −0.79). rs9666607 demonstrated position-specific sensitivity to residue 417. Although K417T (MuPRO ΔΔG = −0.32) and K417R (MuPRO ΔΔG = −0.21) were both classified as 100% neutral with modest destabilization, the alternative substitution K417I was classified as deleterious by five tools (PredictSNP, PhD-SNP, PolyPhen-1, PolyPhen-2, and SNAP; Table 3) and paradoxically showed increased stability (MuPRO ΔΔG = 0.13).

### 3.6 Intercellular regulatory proteins (LCN2)

Analysis of LCN2 revealed no variants that met the established threshold. None of the evaluated SNPs demonstrated a high or moderate deleterious consensus across PredictSNP2.0, indicating an insufficient predicted functional impact to warrant further validation, according to the applied methodology. Consequently, no LCN2 polymorphisms were selected as candidates for experimental validation.

### 3.7 Secreted immune/inflammatory mediators (NTAN1 and RASSF5)

In NTAN1, regulatory variants (rs11075253 C>A, rs1135999 A>C, and rs2280017 C>T) showed moderate deleterious potential (50%, n = 3; CADD, DANN, FunSeq2). Among the missense variants, the rs1136001 polymorphism (G/A, G/C, or G/T at position 15038117) caused H283Y, H283D, or H283A substitutions, respectively, and was considered 100% neutral by both PredictSNP2.0 and PredictSNP1.0 (Table 3). Despite the neutral classification, all three substitutions enabled interaction with PHE286, which is absent in the wild-type structure, and promoted reduced protein stability (MuPRO ΔΔG = −0.8407, −1.0756, and-1.1843, respectively). Position 283 is located in an exposed region (NetSurf, ConSurf) but is not associated with posttranslational modification. The rs1135999 variant (A>C) causes S287A substitution, classified as 50.0% deleterious (n = 3; CADD, DANN, FunSeq2) by PredictSNP2.0, and was considered deleterious by MAPP (Table 3), with decreased stability (MuPRO ΔΔG = −0.8679). The alternative A>G variant causes S287P substitution, classified as 33.3% deleterious (n = 2; CADD, FunSeq2) by PredictSNP2.0 and was considered deleterious by PolyPhen-1 (Table 3), also with decreased stability (MuPRO ΔΔG = −0.9974). Position 287 is characterized as an exposed region (NetSurf, ConSurf), protein kinase C phosphorylation site (NetPhos 0.877), and an O-glycosylation site (NetOGlyc 0.2733).

Analysis of *RASSF5* revealed 14 non-coding variants (rs12124627, rs4845112, rs67223538, rs72755019, rs7530746, rs11119043, rs11119083, rs11589, rs71633591, rs871782, rs3813976, rs4844540, rs6660116 and rs7527917), demonstrating a high deleterious consensus, suggesting widespread regulatory susceptibility across this tumor suppressor locus. Notably, rs4845112 was classified as 100% deleterious by PredictSNP1.0 (Table 3), with substantial protein destabilization (MuPRO ΔΔG = −0.67).

### 3.8 Validation of selected polymorphisms in thyroid nodules

The analysis identified subtype-specific genetic signals with potential utility in the molecular characterization of thyroid lesions, particularly follicular-patterned tumors. Although no polymorphism broadly distinguished malignant from benign states (Tables 4, 5), histological stratification revealed relevant discriminators (Tables 4, 5), suggesting that the evaluated variants may contribute to a more refined assessment of thyroid nodular diseases.

**TABLE 4:** Allelic and genotypic distribution of validated SNPs across histological subtypes: goiter, FA, PTC, and FTC.

Gene and rsID	Allele or genotype	Benign (B)			Malignant (M)			Controls (C)	p-value		HWEp
		G	FA	B	PTC	FTC	M				
ITGA4 rs1143676	A	157	70	227	197	36	233	443	B vs. C = 0.2352* M vs. C = 1.0000* B vs. M = 0.2907*	G vs. C p = 0.802*; G vs. FA p = 1.0000*; G vs. PTC p = 0.882*; G vs. FTC p = 1.0000*; FA vs. C p = 1.0000*; FA vs. PTC p = 1.0000*; FA vs. FTC p = 1.0000*; PTC vs. C p = 1.0000*; PTC vs. FTC p = 1.0000*; FTC vs. C p = 1.0000*	0.06
	G	51	26	77	91	16	107	199			
	AA	60	26	86	70	13	83	158	B vs. C = 0.6588 M vs. C = 1.0000 B vs. M = 0.7218	G vs. C p = 1.0000*; G vs. FA p = 1.0000*; G vs. PTC p = 1.0000*; G vs. FTC p = 1.0000*; FA vs. C p = 1.0000*; FA vs. PTC p = 1.0000*; FA vs. FTC p = 1.0000*; PTC vs. C p = 1.0000*; PTC vs. FTC p = 1.0000*; FTC vs. C p = 1.0000*	
	AG	37	18	55	57	10	67	127			
	GG	7	4	11	17	3	20	36			
ITGAL rs2230433	G	150	66	216	192	42	234	433	B vs. C = 0.6957* M vs. C = 1.0000* B vs. M = 1.0000*	G vs. C p = 1.0000*; G vs. FA p = 1.0000*; G vs. PTC p = 1.0000*; G vs. FTC p = 1.0000*; FA vs. C p = 1.0000*; FA vs. PTC p = 1.0000*; FA vs. FTC p = 1.0000*; PTC vs. C p = 1.0000*; PTC vs. FTC p = 0.507*; FTC vs. C p = 0.444*	0.489
	C	58	30	88	96	10	106	213			
	GG	55	24	79	65	17	82	145	B vs. C = 1.0000 M vs. C = 1.0000 B vs. M = 1.0000	G vs. C p = 1.0000*; G vs. FA p = 1.0000*; G vs. PTC p = 1.0000*; G vs. FTC p = 1.0000*; FA vs. C p = 1.0000*; FA vs. PTC p = 1.0000*; FA vs. FTC p = 1.0000*; PTC vs. C p = 1.0000*; PTC vs. FTC p = 1.0000*; FTC vs. C p = 1.0000*	
	GC	40	18	58	62	8	70	143			
	CC	9	6	15	17	1	18	35			
ITGAM rs1143683	C	166	74	240	240	44	284	519	B vs. C = 1.0000* M vs. C = 0.6867* B vs. M = 0.4677*	G vs. C p = 1.0000*; G vs. FA p = 1.0000*; G vs. PTC p = 1.0000*; G vs. FTC p = 1.0000*; FA vs. C p = 1.0000*; FA vs. PTC p = 1.0000*; FA vs. FTC p = 1.0000*; PTC vs. C p = 1.0000*; PTC vs. FTC p = 1.0000*; FTC vs. C p = 1.0000*	0.106
	T	42	22	64	48	8	56	127			
	CC	66	27	93	101	20	121	214	B vs. C = 0.5607 M vs. C = 1.0000 B vs. M = 0.3153	G vs. C p = 1.0000*; G vs. FA p = 1.0000*; G vs. PTC p = 1.0000*; G vs. FTC p = 1.0000*; FA vs. C p = 1.0000*; FA vs. PTC p = 1.0000*; FA vs. FTC p = 0.285*; PTC vs. C p = 1.0000*; PTC vs. FTC p = 1.0000*; FTC vs. C p = 1.0000*	
	CT	34	20	54	38	4	42	91			
	TT	4	1	5	5	2	7	18			
ICAM1 rs1799969	G	195	88	283	266	46	312	606	B vs. C = 1.0000* M vs. C = 0.5418* B vs. M = 1.0000*	G vs. C p = 1.0000*; G vs. FA p = 1.0000*; G vs. PTC p = 1.0000*; G vs. FTC p = 1.0000*; FA vs. C p = 1.0000*; FA vs. PTC p = 1.0000*; FA vs. FTC p = 1.0000*; PTC vs. C p = 1.0000*; PTC vs. FTC p = 1.0000*; FTC vs. C p = 1.0000*	0.969
	A	13	8	21	22	6	28	38			
	GG	91	40	131	123	20	143	286	B vs. C = 0.8436* M vs. C = 0.2496* B vs. M = 1.0000*	G vs. C p = 1.0000*; G vs. FA p = 1.0000*; G vs. PTC p = 1.0000*; G vs. FTC p = 1.0000*; FA vs. C p = 1.0000*; FA vs. PTC p = 1.0000*; FA vs. FTC p = 1.0000*; PTC vs. C p = 1.0000*; PTC vs. FTC p = 1.0000*; FTC vs. C p = 0.973*	
	GA	13	8	21	20	6	26	34			
	AA	0	0	0	1	0	1	2			
ICAM1 rs5498	A	123	60	183	163	24	187	352	B vs. C = 1.0000* M vs. C = 0.2490* B vs. M = 0.6048*	G vs. C p = 1.0000*; G vs. FA p = 1.0000*; G vs. PTC p = 1.0000*; G vs. FTC p = 1.0000*; FA vs. C p = 1.0000*; FA vs. PTC p = 1.0000*; FA vs. FTC p = 0.590*; PTC vs. C p = 1.0000*; PTC vs. FTC p = 1.0000*; FTC vs. C p = 0.400*	0.814
	G	85	36	121	125	28	153	226			
	AA	38	17	55	45	3	48	119	B vs. C = 1.0000 M vs. C = 0.3453 B vs. M = 0.9294	G vs. C p = 1.0000*; G vs. FA p = 1.0000*; G vs. PTC p = 1.0000*; G vs. FTC p = 0.381*; FA vs. C p = 1.0000*; FA vs. PTC p = 1.0000*; FA vs. FTC p = 0.754*; PTC vs. C p = 1.0000*; PTC vs. FTC p = 1.0000*; FTC vs. C p = 0.233*	
	AG	47	26	73	73	18	91	114			
	GG	19	5	24	26	5	31	56			
MADCAM1 rs3745925	C	167	59	226	231	36	267	497	B vs. C = 0.9603* M vs. C = 1.0000* B vs. M = 0.6663*	G vs. C p = 1.0000*; G vs. FA p = 0.016*; G vs. PTC p = 1.0000*; G vs. FTC p = 0.936*; FA vs. C p = 0.027*; FA vs. PTC p = 0.007*; FA vs. FTC p = 1.0000*; PTC vs. C p = 1.0000*; PTC vs. FTC p = 0.661*; FTC vs. C p = 1.0000*	0.726
	A	41	35	76	55	16	71	141			
	CC	66	16	82	93	14	107	196	B vs. C = 0.6723 M vs. C = 1.0000 B vs. M = 0.5880	G vs. C p = 1.0000*; G vs. FA p = 0.019*; G vs. PTC p = 1.0000*; G vs. FTC p = 0.408*; FA vs. C p = 0.017*; FA vs. PTC p = 0.009*; FA vs. FTC p = 0.905*; PTC vs. C p = 1.0000*; PTC vs. FTC p = 0.434*; FTC vs. C p = 1.0000*	
	CA	35	27	62	45	8	53	105			
	AA	3	4	7	5	4	9	18			
MMP9 rs17576	A	126	59	185	182	33	215	434	B vs. C = 0.4629* M vs. C = 1.0000* B vs. M = 1.0000*	G vs. C p = 1.0000*; G vs. FA p = 1.0000*; G vs. PTC p = 1.0000*; G vs. FTC p = 1.0000*; FA vs. C p = 1.0000*; FA vs. PTC p = 1.0000*; FA vs. FTC p = 1.0000*; PTC vs. C p = 1.0000*; PTC vs. FTC p = 1.0000*; FTC vs. C p = 1.0000*	0.403
	G	72	35	107	96	15	111	202			
	AA	42	19	61	61	12	73	148	B vs. C = 0.7938 M vs. C = 1.0000 B vs. M = 1.0000	G vs. C p = 1.0000*; G vs. FA p = 1.0000*; G vs. PTC p = 1.0000*; G vs. FTC p = 1.0000*; FA vs. C p = 1.0000*; FA vs. PTC p = 1.0000*; FA vs. FTC p = 1.0000*; PTC vs. C p = 1.0000*; PTC vs. FTC p = 1.0000*; FTC vs. C p = 1.0000*	
	AG	42	21	63	60	9	69	138			
	GG	15	7	22	18	3	21	32			
MMP9 rs17577	G	178	80	258	248	48	296	569	B vs. C = 1.0000*M vs. C = 1.0000*B vs. M = 1.0000*	G vs. C p = 1.0000*; G vs. FA p = 1.0000*; G vs. PTC p = 1.0000*; G vs. FTC p = 1.0000*; FA vs. C p = 1.0000*; FA vs. PTC p = 1.0000*; FA vs. FTC p = 1.0000*; PTC vs. C p = 1.0000*; PTC vs. FTC p = 1.0000*; FTC vs. C p = 1.0000*	0.508
	A	28	10	38	34	4	38	73			
	GG	78	35	113	107	22	129	252	B vs. C = 1.0000 M vs. C = 1.0000 B vs. M = 0.5376	G vs. C p = 1.0000*; G vs. FA p = 1.0000*; G vs. PTC p = 1.0000*; G vs. FTC p = 1.0000*; FA vs. C p = 1.0000*; FA vs. PTC p = 1.0000*; FA vs. FTC p = 1.0000*; PTC vs. C p = 1.0000*; PTC vs. FTC p = 1.0000*; FTC vs. C p = 1.0000*	
	GA	22	10	32	34	4	38	65			
	AA	3	0	3	0	0	0	4			

Chi-square test and *Fisher’s exact test. Abbreviations - G, goiter; FA, follicular adenoma; PTC, papillary thyroid cancer; FTC, follicular thyroid cancer; B, benign; M, malignant; C, control; vs, versus; HWEp, Hardy Weinberg equilibrium p-value. All p-values were corrected using the Bonferroni correction.

**TABLE 5:** Clinical and pathological characteristics of thyroid nodule (benign and malignant).

			ITGA4 - rs1143676				ITGAL - rs2230433				ITGAM - 1143683				ICAM1 - rs1799969			
Group	Characteristics		AA	AG	GG	p-value	GG	GC	CC	p-value	CC	CT	TT	p-value	GG	GA	AA	p-value
Control	Gender	Male	24	21	3	0,473	26	21	2	0,1904	38	10	1	0,1619	42	6	1	0,6294
		Female	134	106	33		119	122	33		176	81	17		224	28	1	
Benign	Gender	Male	7	6	3	0,1493	11	5	0	0,228	9	6	1	0,7875*	13	3	0	0,4658
		Female	79	49	8		68	53	15		84	48	4		118	18	0	
Malignant	Gender	Male	15	13	4	0,9688	14	15	4	0,7742	25	6	1	0,6288	30	2	0	0,1139*
		Female	68	54	16		68	56	14		96	36	6		113	24	1	
	Multifocality	Yes	30	18	4	0,3435	32	14	12	**0,0008**	33	14	5	**0,0428**	45	7	0	>0,9999*
		No	52	47	14		49	52	6		86	25	2		97	15	1	
	Capsule	Yes	26	14	2	0,0994	20	19	12	**0,0014**	32	7	3	0,2688	41	1	0	0,0122*
		No	40	40	12		47	33	3		68	22	2		75	16	1	
	CLT	Yes	23	17	6	0,8889	20	21	10	0,1851	28	17	1	0,2015	39	7	0	0,5873*
		No	34	27	7		29	29	5		50	15	3		60	7	1	
	Vascular and lymphatic invasion	Yes	29	23	4	0,3626	26	21	9	0,2443	41	14	1	0,4408	48	8	0	0,8066*
		No	43	38	14		49	39	7		66	23	6		83	11	1	
	Metastasis at diagnosis	Yes	17	9	4	0,4417	15	11	4	0,5904	23	7	0	0,3175	25	5	0	0,5490*
		No	41	38	9		35	41	12		58	25	5		77	10	1	
Control	Gender	Male	22	20	7	0,4754	29	16	3	0,9755	22	18	6	0,6979	39	10	0	>0,9999*
		Female	97	124	49		167	89	15		126	120	26		213	55	4	
Benign	Gender	Male	7	8	1	0,5148	3	12	0	0,0060*	7	6	3	0,8559	14	1	1	0,3605*
		Female	48	65	23		79	50	7		54	57	19		99	31	2	
Malignant	Gender	Male	10	17	5	0,8714	20	10	2	0,9667	20	8	4	0,0602	27	5	0	0,3535*
		Female	38	74	26		87	43	7		53	61	17		102	33	0	
	Multifocality	Yes	13	58	10	**<0.0001**	32	16	4	0,5189	23	20	3	0,2721	42	9	0	0,3184*
		No	35	29	20		71	37	4		49	45	18		82	29	0	
	Capsule	Yes	14	24	4	0,5658	27	12	3	0,7407	19	18	4	0,7604	33	8	0	>0,9999*
		No	30	47	15		57	30	4		44	33	11		72	19	0	
	CLT	Yes	13	21	12	0,4062	35	8	2	0,1271	15	23	5	0,0663	33	12	0	0,4884*
		No	24	33	11		42	24	2		36	21	10		53	13	0	
	Vascular and lymphatic invasion	Yes	21	27	8	0,2730	39	13	3	0,1533	27	22	5	0,4677	47	9	0	0,1586*
		No	24	53	18		53	37	5		38	39	14		67	25	0	
	Metastasis at diagnosis	Yes	9	16	5	0,8671	23	5	2	0,0841	16	10	3	0,5548	23	7	0	0,8010
		No	30	42	16		53	32	2		37	37	11		67	18	0	

CLT, chronic lymphocyte thyroiditis.

Bold values indicates p < 0.05.

*Fisher's exact test.

The *MADCAM1* rs3745925 variant emerged as the most informative marker, significantly distinguishing follicular adenoma from controls (p = 0.017, OR: 3.15; 95% CI: 1.631–6.053), goiter (p = 0.019, OR: 3.18; 95% CI: 1.49–6.85), and PTC (p = 0.009, OR: 3.49, 95% CI: 1.728–7.092), underscoring its potential value as an auxiliary biomarker in the differential characterization of thyroid nodular lesions. Additionally, ITGAM rs1143683, ITGAL rs2230433, and ICAM1 rs5498 were associated with tumor multifocality (p = 0.0428, p = 0.0008, and p < 0.0001, respectively).

## 4 Discussion

This integrated *in silico* and clinical study provides insights into the potential contribution of variations in adhesion- and immune-related genes to the molecular characterization of thyroid nodular diseases. Across the immune adhesion and extracellular matrix remodeling networks analyzed, bioinformatic prioritization revealed a clear gradient of predicted functional disruption, with many variants concentrated in regulatory regions rather than being limited to protein-coding changes. Several loci showed recurrent deleterious predictions across independent algorithms, suggesting that disease susceptibility in this dataset is likely shaped by both transcriptional/splicing regulation and structural protein effects.

This was particularly evident for *ICAM2*, *JAM3*, *F11R*, *ITGA4*, *ITGAM*, *ITGB2*, *TIMP2*, *RASSF5*, and *CD44*, which carried multiple non-coding variants with a consistent deleterious consensus, supporting the idea that altered gene regulation may be a major driver of biological impact. Simultaneously, selected missense substitutions displayed functionally meaningful consequences, including reduced stability, altered residue interactions, exposure at functionally relevant sites, and disruption of cleavage or modification motifs. In this context, the integrative use of consensus predictors, stability estimators, conservation analysis, and structure-based annotations is essential to distinguish variants with likely biological relevance from those appearing neutral in isolated prediction tools.

Among the coding changes, the most compelling signals were observed in *ICAM1*, *ICAM3*, *MADCAM1*, *PECAM1*, *VCAM1*, *MMP9*, *THBS1*, *NTAN1*, and *F11R*, where the cumulative burden of deleterious substitutions was the highest. Variants such as *MMP9* rs17576, *THBS1* rs2228261 and rs2292305, *ICAM3* rs2304237 and rs7258015, and *CD44* rs1071695 and rs9666607 illustrate how bioinformatics can reveal functionally plausible candidates, even when prediction outputs are not uniformly concordant. Importantly, several substitutions classified as neutral by some algorithms still showed marked destabilization or loss of residue contacts, underscoring the value of integrating multiple complementary approaches rather than relying on any single method alone.

By systematically screening 407,812 polymorphisms across 22 candidate genes, we prioritized 133 variants with high predicted functional impact, eight of which were selected for clinical validation in a cohort of 648 individuals. Among these, *MADCAM1* rs3745925 emerged as the only polymorphism that showed a consistent and statistically significant clinical signal. This variant distinguished follicular adenoma from controls (p = 0.017, OR: 3.15; 95% CI: 1.631–6.053), goiter (p = 0.019, OR: 3.18; 95% CI: 1.49–6.85), and papillary thyroid carcinoma (p = 0.009, OR: 3.49; 95% CI: 1.728–7.092). Notably, this association remained robust after Bonferroni correction for multiple comparisons, supporting its potential value as an auxiliary marker for the differential characterization of follicular-patterned thyroid lesions. MAdCAM-1 regulates lymphocyte homing to mucosal tissues (Keir et al., 2021; Iizuka et al., 2000; Kuhbandner et al., 2019), and although its role in thyroid biology is poorly defined, our results suggest that genetic variation at this locus may influence follicular cell proliferation and early tumorigenesis in thyroid tissues.

Additionally, *ITGAM* rs1143683, *ITGAL* rs2230433, and *ICAM1* rs5498 were associated with tumor multifocality (p = 0.0428, p = 0.0008, and p < 0.0001, respectively); however, these associations did not survive Bonferroni correction and should therefore be interpreted as exploratory. In contrast, variants in *ITGA4*, *ITGAL*, and *MMP9* were not significantly associated with this cohort. This does not discount their biological relevance in thyroid cancer progression but suggests that the effects of these common polymorphisms may be modest or context-dependent, requiring larger cohorts or integrative multi-omics approaches for detection.

Several limitations should be considered when interpreting the findings. First, the sample size of certain histological subgroups, particularly FTC, was modest and may have limited statistical power. Second, as a single-center study, external validation in independent populations is required to strengthen generalizability. Third, functional assays are required to elucidate the mechanistic consequences of the identified variants and explore their predicted impacts on protein structure, stability, and post-translational modifications. Fourth, given the exploratory nature of this study, the observed associations should be viewed as hypothesis-generating only. Finally, while we selected eight SNPs for clinical validation based on a multi-step prioritization strategy, other potentially relevant polymorphisms from the 133 prioritized variants may also merit further investigation.

Despite these limitations, this study had several strengths. The integrated approach combining systematic bioinformatics prioritization with clinical validation in a well-characterized cohort of 648 individuals provides a model for discovering candidate genetic markers of thyroid nodular disease. The identification of *MADCAM1* rs3745925 as a variant that distinguishes follicular adenoma from goiter, PTC, and controls represents a specific, testable finding that can be directly examined in future studies. Moreover, the associations observed for *ITGAM* rs1143683, *ITGAL* rs2230433, and *ICAM1* rs5498 with tumor multifocality generate new hypotheses regarding the role of adhesion and immune pathways in thyroid tumor pathobiology. Collectively, this study establishes a foundation for larger association studies and functional experiments aimed at clarifying the biological and clinical relevance of these genetic variants.

## 5 Conclusion

This exploratory study identified specific germline polymorphisms in cell adhesion- and immune-related genes associated with distinct clinicopathological features of thyroid nodular diseases. Notably, the association of *MADCAM1* rs3745925 with follicular adenoma and follicular-patterned lesions remained robust after stringent correction for multiple comparisons, positioning this variant as a priority candidate for future studies. The additional associations of *ITGAM* rs1143683, *ITGAL* rs2230433, and *ICAM1* rs5498 with tumor multifocality, although not surviving Bonferroni correction, offer preliminary hypotheses that warrant further investigation. Collectively, these findings support the involvement of adhesion and immune pathways in thyroid tumor biology and demonstrate the feasibility of integrating computational variant prioritization with targeted clinical validation of the results. Independent replication in larger, multicenter cohorts and functional studies is needed to clarify the biological and clinical relevance of these variants.

## Data Availability

The original contributions presented in the study are included in the article/Supplementary Material, further inquiries can be directed to the corresponding author.
