# Supplementary material for: A bioinformatics pipeline for identifying clinically relevant immune and adhesion biomarkers in thyroid risk stratification
Source: Front Mol Biosci. 2026 Jun 23;13:1814666. doi: 10.3389/fmolb.2026.1814666 (PMC13337396; doi:10.3389/fmolb.2026.1814666)
Supplement: Supplementary file 1 [file Table1.doc]

**Supplementary Tables**

Supplementary Table 1: Description of the selected polymorphisms for validation in thyroid nodules coorte.

| **Gene** | **SNP ID** | **Chr#**  **Location** | **Nucleotide Exchange** | **Assay ID** | **Context Sequence** | **Vic** | **Fam** | **MAF ABraOM** |
| --- | --- | --- | --- | --- | --- | --- | --- | --- |
| ITGA4 | rs1143676 | Chr.2  181530618 | G/A | C___1276216_1_ | ATGCAGACCTTGAAAGGCATAGTCC**[A/G]**GTTCTTGTCCAAGACTGATAAGAGG | A | G | 0.721605 |
| ITGAL | rs2230433 | Chr.16  30506720 | G/C | C__11789692_10 | TCCCTGATCATCCCCCACAGATCCA**[G/C]**AGCCCTGCGTCTAACTGCTTTTGCC | G | C | 0.310418 |
| ITGAM | rs1143683 | Chr.16  31325567 | C/T | C__11388882_20 | GCCTCCTCCACCGAAGTGTCTGGGG**[T/C]**CTTGAAGAGCACCAGCTGCAGCATA | T | C | 0.227156 |
| ICAM1 | rs1799969 | Chr.19  10284116 | G/A | C___8726331_10 | GGGGACCGTGGTCTGTTCCCTGGAC**[A/G]**GGCTGTTCCCAGTCTCGGAGGCCCA | A | G | 0.08497 |
| ICAM1 | rs5498 | Chr.19  10285007 | A/G | C___8726337_40 | GAGCACTCAAGGGGAGGTCACCCGC**[A/G]**AGGTGACCGTGAATGTGCTCTGTGA | A | G | 0.430401 |
| MADCAM1 | rs3745925 | Chr.19  501900 | C/A | C__27472568_10 | CGCCCTGAGATCTCCCAGGCTGGGC**[C/A]**CACGCAGGGAGAAGTGATCCCAACA | C | A | 0.227156 |
| MMP9 | rs17576 | Chr.20  46011586 | A/G | C__11655953_10 | CTCCTCGCCCCAGGACTCTACACCC**[A/G]**GGACGGCAATGCTGATGGGAAACCC | A | G | 0.339026 |
| MMP9 | rs17577 | Chr.20  46014472 | G/A | C__11655948_1_ | GACACGCACGACGTCTTCCAGTACC**[A/G]**AGGTGAGGGCTGAGGAGGATCCCTT | A | G | 0.129804 |

*Chr#: Chromossome; ID: Identification; MAF: minor allele frequency*

Supplementary Table 2: In silico analysis data of most deleterious SNPs in twenty-two evaluated genes.

|  | **CHR** | **POS** | **rsID** | **R** | **P** |  | **PredictSNP2.0** | **CADD** | **DANN** | **FATHMM-MLK** | **FUNSEQ2** | **GWAVA** | **%D** |
| --- | --- | --- | --- | --- | --- | --- | --- | --- | --- | --- | --- | --- | --- |
| ICAM1 | 19 | 10286547 | rs923366 | C | A | UTR3 | N | N | N | N | D | D | 33,3 |
|  | 19 | 10286547 | rs923366 | C | T | UTR3 | N | N | N | N | D | D | 33,3 |
|  | 19 | 10285007 | rs5498 | A | G | exonic | N | N | N | N | N | D | 16,7 |
| ICAM2 | 17 | 64010895 | rs4968683 | A | C | intronic | D | D | D | N | N | N | 50,0 |
|  | 17 | 64010895 | rs4968683 | A | G | intronic | D | D | D | N | N | N | 50,0 |
|  | 17 | 64010895 | rs4968683 | A | T | intronic | D | D | D | N | N | N | 50,0 |
|  | 17 | 64011037 | rs4968605 | A | C | intronic | D | D | D | ? | N | N | 50,0 |
|  | 17 | 64011037 | rs4968605 | A | T | intronic | D | D | D | ? | N | N | 50,0 |
|  | 17 | 64020486 | rs16947352 | C | T | UTR5 | N | D | D | N | D | N | 50,0 |
| ICAM3 | 19 | 10337984 | rs7250300 | C | A | intronic | D | D | D | ? | N | N | 50,0 |
|  | 19 | 10335892 | rs2304237 | T | C | exonic | N | N | N | N | D | D | 33,3 |
|  | 19 | 10335892 | rs2304237 | T | G | exonic | N | N | N | N | D | D | 33,3 |
|  | 19 | 10338682 | rs7258015 | T | C | exonic | N | N | N | N | N | D | 16,7 |
| MADCAM1 | 19 | 495722 | rs758503 | G | A | upstream | N | N | N | N | D | D | 33,3 |
|  | 19 | 495722 | rs758503 | G | C | upstream | N | N | N | N | D | D | 33,3 |
|  | 19 | 495722 | rs758503 | G | T | upstream | N | N | N | N | D | D | 33,3 |
|  | 19 | 501900 | rs3745925 | C | A | exonic | N | N | N | N | N | N | 0,0 |
|  | 19 | 501900 | rs3745925 | C | G | exonic | N | N | N | N | N | N | 0,0 |
| PECAM1 | 17 | 64322512 | rs9902260 | C | A | UTR3 | D | D | N | D | D | N | 66,7 |
|  | 17 | 64322512 | rs9902260 | C | G | UTR3 | D | D | N | D | D | N | 66,7 |
|  | 17 | 64322512 | rs9902260 | C | T | UTR3 | D | D | N | D | D | N | 66,7 |
|  | 17 | 64327030 | rs1470453 | T | A | intronic | D | D | D | ? | D | N | 66,7 |
|  | 17 | 64319386 | rs12941698 | T | A | downstream | D | D | D | ? | N | N | 50,0 |
|  | 17 | 64319386 | rs12941698 | T | C | downstream | D | D | D | ? | N | N | 50,0 |
| VCAM1 | 1 | 100725310 | rs3917010 | A | G | intronic | D | D | D | D | D | D | 100,0 |
|  | 1 | 100724617 | rs2392221 | C | A | intronic | D | D | N | D | D | N | 66,7 |
|  | 1 | 100725310 | rs3917010 | A | C | intronic | N | N | N | D | D | D | 50,0 |
|  | 1 | 100724617 | rs2392221 | C | T | intronic | N | D | N | D | D | N | 50,0 |
| JAM3 | 11 | 134086563 | rs621698 | G | A | intronic | D | D | D | D | D | N | 83,3 |
|  | 11 | 134086693 | rs659196 | T | A | intronic | D | D | D | D | D | N | 83,3 |
|  | 11 | 134086693 | rs659196 | T | C | intronic | D | D | D | D | D | N | 83,3 |
|  | 11 | 134086693 | rs659196 | T | G | intronic | D | D | D | D | D | N | 83,3 |
|  | 11 | 134148761 | rs610382 | T | A | intronic | D | D | D | D | D | N | 83,3 |
|  | 11 | 134148761 | rs610382 | T | C | intronic | D | D | D | D | D | N | 83,3 |
|  | 11 | 134150006 | rs7936421 | T | C | UTR3 | D | D | D | D | D | N | 83,3 |
|  | 11 | 134086563 | rs621698 | G | C | intronic | D | D | ? | D | D | N | 66,7 |
|  | 11 | 134071516 | rs595102 | G | A | intronic | D | D | N | D | D | N | 66,7 |
|  | 11 | 134092311 | rs11223686 | C | A | intronic | D | D | D | D | N | N | 66,7 |
|  | 11 | 134103212 | rs1143809 | A | C | intronic | D | D | D | D | N | N | 66,7 |
|  | 11 | 134103212 | rs1143809 | A | T | intronic | D | D | D | D | N | N | 66,7 |
|  | 11 | 134115603 | rs470966 | T | C | intronic | D | D | D | D | N | N | 66,7 |
|  | 11 | 134120269 | rs12287552 | C | T | intronic | D | D | N | D | D | N | 66,7 |
|  | 11 | 134123361 | rs470846 | A | G | intronic | D | D | N | D | D | N | 66,7 |
|  | 11 | 134132973 | rs470417 | C | T | intronic | D | D | D | D | N | N | 66,7 |
|  | 11 | 134085430 | rs647837 | A | G | intronic | D | D | D | N | N | N | 50,0 |
|  | 11 | 134085430 | rs647837 | A | T | intronic | D | D | D | N | N | N | 50,0 |
|  | 11 | 134092311 | rs11223686 | C | G | intronic | D | D | N | D | N | N | 50,0 |
|  | 11 | 134092311 | rs11223686 | C | T | intronic | D | D | ? | D | N | N | 50,0 |
|  | 11 | 134102264 | rs470456 | G | T | intronic | D | D | D | N | N | N | 50,0 |
|  | 11 | 134104601 | rs1143811 | T | A | intronic | D | D | D | N | N | N | 50,0 |
|  | 11 | 134104601 | rs1143811 | T | G | intronic | D | D | D | N | N | N | 50,0 |
|  | 11 | 134106767 | rs637517 | C | G | intronic | D | D | D | ? | N | N | 50,0 |
|  | 11 | 134124288 | rs7101690 | T | A | intronic | D | D | N | D | N | N | 50,0 |
|  | 11 | 134124288 | rs7101690 | T | G | intronic | D | D | N | D | N | N | 50,0 |
|  | 11 | 134125356 | rs11223703 | G | A | intronic | N | D | N | N | D | D | 50,0 |
|  | 11 | 134134878 | rs11223709 | T | C | intronic | D | D | D | ? | N | N | 50,0 |
|  | 11 | 134134878 | rs11223709 | T | G | intronic | D | D | D | ? | N | N | 50,0 |
|  | 11 | 134139965 | rs2276266 | T | C | intronic | N | D | D | N | D | N | 50,0 |
|  | 11 | 134139965 | rs2276266 | T | G | intronic | N | D | D | N | D | N | 50,0 |
|  | 11 | 134148850 | rs610829 | G | T | intronic | D | D | D | ? | N | N | 50,0 |
| F11R | 1 | 161021086 | rs28365979 | C | T | UTR5 | D | D | D | D | D | D | 100,0 |
|  | 1 | 161021085 | rs28365980 | T | C | UTR5 | D | D | N | D | D | D | 83,3 |
|  | 1 | 161021086 | rs28365979 | C | A | UTR5 | D | D | N | D | D | D | 83,3 |
|  | 1 | 161021086 | rs28365979 | C | G | UTR5 | D | D | N | D | D | D | 83,3 |
|  | 1 | 161021088 | rs28365978 | C | G | UTR5 | D | D | N | D | D | D | 83,3 |
|  | 1 | 161021088 | rs28365978 | C | T | UTR5 | D | D | ? | D | D | D | 83,3 |
|  | 1 | 161021116 | rs2481085 | G | A | UTR5 | D | D | D | D | D | N | 83,3 |
|  | 1 | 161001356 | rs790055 | A | C | intronic | D | D | N | D | D | N | 66,7 |
|  | 1 | 161001356 | rs790055 | A | G | intronic | D | D | N | D | D | N | 66,7 |
|  | 1 | 161001356 | rs790055 | A | T | intronic | D | D | N | D | D | N | 66,7 |
|  | 1 | 161012467 | rs11807930 | A | G | intronic | D | D | D | D | N | N | 66,7 |
|  | 1 | 161012467 | rs11807930 | A | T | intronic | D | D | D | D | N | N | 66,7 |
|  | 1 | 161008964 | rs2988726 | T | C | intronic | N | N | D | ? | D | D | 50,0 |
|  | 1 | 161019824 | rs2481084 | T | C | intronic | N | D | D | N | D | N | 50,0 |

*CHR: Chromossome; R: Reference Allele; P: Polymorphic allele; %D: Deleterious percentage; D: Deleterious; N: Neutral*

|  | **CHR** | **POS** | **rsID** | **R** | **P** |  | **PredictSNP**  **2.0** | **CADD** | **DANN** | **FATHMM-MLK** | **FUNSEQ2** | **GWAVA** | **%D** |
| --- | --- | --- | --- | --- | --- | --- | --- | --- | --- | --- | --- | --- | --- |
| ITGA4 | 2 | 181485720 | rs155102 | A | G | intronic | D | D | D | D | D | D | 100,0 |
|  | 2 | 181485762 | rs17290693 | T | C | intronic | D | D | D | D | D | D | 100,0 |
|  | 2 | 181461180 | rs3770138 | C | A | intronic | D | D | N | D | D | D | 83,3 |
|  | 2 | 181461180 | rs3770138 | C | T | intronic | D | D | N | D | D | D | 83,3 |
|  | 2 | 181495455 | rs2305586 | T | A | intronic | D | D | N | D | D | D | 83,3 |
|  | 2 | 181521228 | rs62191453 | A | C | intronic | D | D | D | D | D | N | 83,3 |
|  | 2 | 181521228 | rs62191453 | A | G | intronic | D | D | D | D | D | N | 83,3 |
|  | 2 | 181523901 | rs3770109 | A | G | intronic | D | D | N | D | D | D | 83,3 |
|  | 2 | 181457493 | rs113276800 | C | A | UTR5 | N | D | D | N | D | D | 66,7 |
|  | 2 | 181457493 | rs113276800 | C | T | UTR5 | N | D | D | N | D | D | 66,7 |
|  | 2 | 181461180 | rs3770138 | C | G | intronic | N | D | N | D | D | D | 66,7 |
|  | 2 | 181490784 | rs4667308 | G | A | intronic | D | D | D | ? | D | N | 66,7 |
|  | 2 | 181493728 | rs16867433 | A | T | intronic | D | D | D | D | N | N | 66,7 |
|  | 2 | 181524837 | rs3770108 | T | A | intronic | D | D | D | D | N | N | 66,7 |
|  | 2 | 181524837 | rs3770108 | T | C | intronic | D | D | D | D | N | N | 66,7 |
|  | 2 | 181482345 | rs2305588 | T | A | intronic | D | D | N | ? | D | N | 50,0 |
|  | 2 | 181499916 | rs3770116 | G | T | intronic | N | D | D | N | D | N | 50,0 |
|  | 2 | 181533215 | rs6748657 | C | A | intronic | N | D | D | N | D | N | 50,0 |
|  | 2 | 181535035 | rs16867438 | G | A | intronic | D | D | D | ? | N | N | 50,0 |
|  | 2 | 181536389 | rs10490691 | C | G | UTR3 | D | D | N | D | ? | N | 50,0 |
|  | 2 | 181536389 | rs10490691 | C | T | UTR3 | D | D | N | D | ? | N | 50,0 |
|  | 2 | 181530618 | rs1143676 | G | A | exonic | N | N | N | N | N | D | 16,7 |
|  | 2 | 181530618 | rs1143676 | G | T | exonic | N | N | N | N | N | D | 16,7 |
|  | 2 | 181534370 | rs7562325 | T | C | exonic | N | D | N | N | N | N | 16,7 |
|  | 2 | 181534370 | rs7562325 | T | A | exonic | N | N | N | N | N | N | 0,0 |
|  | 2 | 181534370 | rs7562325 | T | G | exonic | N | N | N | N | N | N | 0,0 |
| ITGAD | 16 | 31393250 | rs8050500 | T | A | upstream | D | D | D | ? | D | D | 83,3 |
|  | 16 | 31393250 | rs8050500 | T | C | upstream | D | D | D | N | D | D | 83,3 |
|  | 16 | 31393250 | rs8050500 | T | G | upstream | D | D | D | ? | D | D | 83,3 |
| ITGAL | 16 | 30471173 | rs11150589 | T | A | intergenic | D | D | D | D | D | N | 83,3 |
|  | 16 | 30471173 | rs11150589 | T | C | intergenic | D | D | D | D | D | N | 83,3 |
|  | 16 | 30471173 | rs11150589 | T | G | intergenic | D | D | D | D | D | N | 83,3 |
|  | 16 | 30471482 | rs12716977 | T | A | intergenic | D | D | D | D | N | N | 66,7 |
|  | 16 | 30471482 | rs12716977 | T | C | intergenic | D | D | D | D | N | N | 66,7 |
|  | 16 | 30471482 | rs12716977 | T | G | intergenic | D | D | D | D | N | N | 66,7 |
|  | 16 | 30474072 | rs11574938 | G | A | intronic | D | D | D | ? | D | N | 66,7 |
|  | 16 | 30474072 | rs11574938 | G | T | intronic | D | D | D | ? | D | N | 66,7 |
|  | 16 | 30471219 | rs12598978 | T | A | intergenic | N | D | D | N | D | N | 50,0 |
|  | 16 | 30471219 | rs12598978 | T | G | intergenic | N | D | D | N | D | N | 50,0 |
|  | 16 | 30474072 | rs11574938 | G | C | intronic | D | D | N | ? | D | N | 50,0 |
|  | 16 | 30481679 | rs11574941 | G | A | intronic | N | D | D | N | D | N | 50,0 |
|  | 16 | 30499250 | rs1557672 | C | A | intronic | D | D | D | ? | N | N | 50,0 |
|  | 16 | 30502782 | rs4341759 | G | A | intronic | D | D | N | D | N | N | 50,0 |
|  | 16 | 30506720 | rs2230433 | G | C | exonic | N | N | N | N | N | ? | 0,0 |
| ITGAM | 16 | 31271843 | rs35314490 | G | A | intronic | D | D | N | D | D | N | 66,7 |
|  | 16 | 31271843 | rs35314490 | G | T | intronic | D | D | N | D | D | N | 66,7 |
|  | 16 | 31291880 | rs9940397 | T | C | intronic | D | D | D | D | N | N | 66,7 |
|  | 16 | 31319686 | rs4889647 | G | T | intronic | D | D | D | D | N | N | 66,7 |
|  | 16 | 31276800 | rs9939679 | A | T | intronic | N | N | D | N | D | D | 50,0 |
|  | 16 | 31279739 | rs140615691 | T | G | intronic | D | D | D | ? | N | N | 50,0 |
|  | 16 | 31294999 | rs9928749 | T | A | intronic | D | D | D | ? | N | N | 50,0 |
|  | 16 | 31294999 | rs9928749 | T | C | intronic | D | D | D | ? | N | N | 50,0 |
|  | 16 | 31307962 | rs7198598 | G | A | intronic | D | D | D | ? | N | N | 50,0 |
|  | 16 | 31315266 | rs11865746 | T | G | intronic | D | D | D | ? | N | N | 50,0 |
|  | 16 | 31319686 | rs4889647 | G | A | intronic | D | D | N | D | N | N | 50,0 |
|  | 16 | 31319686 | rs4889647 | G | C | intronic | D | D | ? | D | N | N | 50,0 |
|  | 16 | 31319816 | rs11864503 | T | C | intronic | D | D | D | ? | N | N | 50,0 |
|  | 16 | 31332065 | rs9933520 | A | G | UTR3 | N | D | D | N | ? | D | 50,0 |
|  | 16 | 31331684 | rs1143678 | C | T | exonic | N | N | N | N | N | D | 16,7 |
|  | 16 | 31325567 | rs1143683 | C | T | exonic | N | N | N | N | N | N | 0,0 |
| ITGB2 | 21 | 44910713 | rs2280965 | C | T | intronic | D | D | D | ? | D | D | 83,3 |
|  | 21 | 44895992 | rs2838727 | C | T | intronic | D | N | D | D | D | N | 66,7 |
|  | 21 | 44905141 | rs59498405 | C | T | intronic | D | D | D | N | D | N | 66,7 |
|  | 21 | 44907111 | rs2072703 | C | A | intronic | D | D | D | ? | D | N | 66,7 |
|  | 21 | 44907111 | rs2072703 | C | T | intronic | D | D | D | ? | D | N | 66,7 |
|  | 21 | 44907372 | rs2072702 | G | A | intronic | N | D | D | N | D | N | 50,0 |
|  | 21 | 44916633 | rs7281781 | T | A | intronic | D | D | D | N | ? | N | 50,0 |

*CHR: Chromossome; R: Reference Allele; P: Polymorphic allele; %D: Deleterious percentage; D: Deleterious; N: Neutral*

|  | **#CHR** | **POS** | **ID** | **R** | **P** |  | | **PredictSNP2.0** | **CADD** | **DANN** | **FATHMM-MLK** | **FUNSEQ2** | **GWAVA** | **%D** |
| --- | --- | --- | --- | --- | --- | --- | --- | --- | --- | --- | --- | --- | --- | --- |
| MMP9 | 20 | 46015131 | rs3918262 | A | G | intronic | | D | D | D | D | D | D | 100,0 |
|  | 20 | 46015131 | rs3918262 | A | T | intronic | | D | D | D | D | D | D | 100,0 |
|  | 20 | 46016514 | rs9509 | T | C | UTR3 | | D | D | D | D | D | D | 100,0 |
|  | 20 | 46011053 | rs2274755 | G | T | intronic | | D | D | D | D | D | N | 83,3 |
|  | 20 | 46016371 | rs20544 | C | G | UTR3 | | D | D | N | D | D | N | 66,7 |
|  | 20 | 46016371 | rs20544 | C | T | UTR3 | | D | D | N | D | D | N | 66,7 |
|  | 20 | 46006977 | rs3918240 | C | T | intergenic | | D | D | N | D | ? | N | 50,0 |
|  | 20 | 46010872 | rs3918253 | C | T | intronic | | D | D | D | ? | ? | N | 50,0 |
|  | 20 | 46011586 | rs17576 | A | C | exonic | | N | D | N | N | D | D | 50,0 |
|  | 20 | 46011586 | rs17576 | A | G | exonic | | N | D | N | N | D | D | 50,0 |
|  | 20 | 46011586 | rs17576 | A | T | exonic | | N | D | N | N | D | D | 50,0 |
|  | 20 | 46013767 | rs2250889 | G | A | exonic | | N | N | D | N | D | D | 50,0 |
|  | 20 | 46013767 | rs2250889 | G | C | exonic | | N | N | N | N | D | D | 33,3 |
|  | 20 | 46013767 | rs2250889 | G | T | exonic | | N | N | N | N | D | D | 33,3 |
|  | 20 | 46014472 | rs17577 | G | A | exonic | | N | N | N | N | N | D | 16,7 |
|  | 20 | 46014472 | rs17577 | G | C | exonic | | N | N | N | N | N | D | 16,7 |
| TIMP1 | X | 47587254 | rs6609534 | G | A | intronic | | N | N | D | ? | D | D | 50,0 |
|  | X | 47587254 | rs6609534 | G | T | intronic | | N | N | D | ? | D | D | 50,0 |
|  | X | 47585586 | rs4898 | T | A | exonic | | N | N | D | N | N | N | 16,7 |
|  | X | 47585586 | rs4898 | T | C | exonic | | N | N | N | N | N | N | 0,0 |
| TIMP2 | 17 | 78879819 | rs7222198 | T | A | intronic | | D | D | N | ? | D | D | 66,7 |
|  | 17 | 78901892 | rs4789936 | C | T | UTR5 | | D | D | D | D | ? | N | 66,7 |
|  | 17 | 78919376 | rs12452379 | C | A | intronic | | D | D | D | D | N | N | 66,7 |
|  | 17 | 78925567 | rs8179096 | G | A | upstream | | D | D | D | ? | D | N | 66,7 |
|  | 17 | 78879654 | rs72849591 | G | A | intronic | | D | D | D | ? | ? | N | 50,0 |
|  | 17 | 78879819 | rs7222198 | T | C | intronic | | N | D | N | N | D | D | 50,0 |
|  | 17 | 78879819 | rs7222198 | T | G | intronic | | N | D | N | ? | D | D | 50,0 |
|  | 17 | 78898398 | rs9912418 | T | A | intronic | | N | D | D | N | D | N | 50,0 |
|  | 17 | 78901892 | rs4789936 | C | G | UTR5 | | D | D | N | D | ? | N | 50,0 |
| THBS1 | 15 | 39581083 | rs1478605 | G | A | UTR5 | | D | D | D | D | D | D | 100,0 |
|  | 15 | 39580382 | rs2664139 | T | C | upstream | | N | N | N | D | D | D | 50,0 |
|  | 15 | 39584211 | rs7170682 | C | T | intronic | | N | D | D | N | D | N | 50,0 |
|  | 15 | 39588157 | rs2228261 | C | A | exonic | | N | N | N | N | D | D | 33,3 |
|  | 15 | 39588157 | rs2228261 | C | G | exonic | | N | N | N | N | D | D | 33,3 |
|  | 15 | 39588621 | rs2292305 | A | C | exonic | | N | N | N | N | D | ? | 16,7 |
|  | 15 | 39588621 | rs2292305 | A | G | exonic | | N | N | N | N | D | ? | 16,7 |
|  | 15 | 39588157 | rs2228261 | C | T | exonic | | N | N | N | N | N | N | 0,0 |
| CD44 | 11 | 35223340 | rs10836343 | T | C | | intronic | D | D | D | D | D | N | 83,3 |
|  | 11 | 35139741 | rs12365962 | G | A | | intronic | D | D | D | N | D | N | 66,7 |
|  | 11 | 35141567 | rs11033008 | T | C | | intronic | D | D | D | ? | D | N | 66,7 |
|  | 11 | 35145812 | rs11033010 | G | A | | intronic | D | D | N | D | D | N | 66,7 |
|  | 11 | 35148308 | rs353624 | G | A | | intronic | D | D | D | ? | D | N | 66,7 |
|  | 11 | 35152589 | rs353620 | A | C | | intronic | D | D | N | D | D | N | 66,7 |
|  | 11 | 35152589 | rs353620 | A | G | | intronic | D | D | N | D | D | N | 66,7 |
|  | 11 | 35152589 | rs353620 | A | T | | intronic | D | D | N | D | D | N | 66,7 |
|  | 11 | 35160066 | rs353644 | T | A | | intronic | D | D | D | D | ? | N | 66,7 |
|  | 11 | 35160066 | rs353644 | T | G | | intronic | D | D | D | D | ? | N | 66,7 |
|  | 11 | 35166742 | rs353629 | C | T | | intronic | D | D | D | ? | D | N | 66,7 |
|  | 11 | 35166960 | rs353628 | G | A | | intronic | N | D | D | N | D | D | 66,7 |
|  | 11 | 35170667 | rs3794110 | G | A | | intronic | D | D | D | ? | D | N | 66,7 |
|  | 11 | 35196092 | rs11033022 | T | C | | intronic | D | D | D | ? | D | N | 66,7 |
|  | 11 | 35196198 | rs10836340 | T | A | | intronic | D | D | D | N | D | N | 66,7 |
|  | 11 | 35196198 | rs10836340 | T | C | | intronic | D | D | D | N | D | N | 66,7 |
|  | 11 | 35196198 | rs10836340 | T | G | | intronic | D | D | D | N | D | N | 66,7 |
|  | 11 | 35227233 | rs4756199 | T | G | | intronic | D | D | D | D | ? | N | 66,7 |
|  | 11 | 35160066 | rs353644 | T | C | | intronic | D | D | D | ? | ? | N | 50,0 |
|  | 11 | 35166742 | rs353629 | C | G | | intronic | D | D | N | ? | D | N | 50,0 |
|  | 11 | 35139876 | rs12362973 | A | G | | intronic | N | D | D | N | D | N | 50,0 |
|  | 11 | 35140123 | rs353558 | T | A | | intronic | N | D | D | N | D | N | 50,0 |
|  | 11 | 35140123 | rs353558 | T | C | | intronic | N | D | D | N | D | N | 50,0 |
|  | 11 | 35140123 | rs353558 | T | G | | intronic | N | D | D | N | D | N | 50,0 |
|  | 11 | 35142561 | rs2553808 | C | T | | intronic | N | N | D | N | D | D | 50,0 |
|  | 11 | 35157162 | rs1981154 | A | T | | intronic | D | D | D | N | ? | N | 50,0 |
|  | 11 | 35158104 | rs353612 | G | A | | intronic | N | D | D | N | ? | D | 50,0 |
|  | 11 | 35158104 | rs353612 | G | C | | intronic | N | D | N | N | D | D | 50,0 |
|  | 11 | 35167204 | rs353627 | C | T | | intronic | D | D | ? | ? | D | N | 50,0 |
|  | 11 | 35170732 | rs3794109 | A | C | | intronic | N | D | D | N | D | N | 50,0 |
|  | 11 | 35170732 | rs3794109 | A | G | | intronic | N | D | D | N | D | N | 50,0 |
|  | 11 | 35170732 | rs3794109 | A | T | | intronic | D | D | D | N | ? | N | 50,0 |
|  | 11 | 35219682 | rs2295756 | T | A | | intronic | D | D | N | ? | D | N | 50,0 |
|  | 11 | 35221360 | rs10734436 | G | A | | intronic | D | D | N | ? | D | N | 50,0 |
|  | 11 | 35221360 | rs10734436 | G | C | | intronic | D | D | N | ? | D | N | 50,0 |
|  | 11 | 35221360 | rs10734436 | G | T | | intronic | D | D | N | ? | D | N | 50,0 |
|  | 11 | 35226512 | rs74938975 | A | G | | intronic | D | D | ? | ? | D | N | 50,0 |
|  | 11 | 35180295 | rs1071695 | C | A | | exonic | N | N | N | N | D | D | 33,3 |
|  | 11 | 35180295 | rs1071695 | C | G | | exonic | N | N | N | N | D | D | 33,3 |
|  | 11 | 35204608 | rs9666607 | A | C | | exonic | N | N | N | N | D | ? | 16,7 |
|  | 11 | 35204608 | rs9666607 | A | G | | exonic | N | N | N | N | D | ? | 16,7 |
|  | 11 | 35204608 | rs9666607 | A | T | | exonic | N | N | N | N | D | ? | 16,7 |
|  | 11 | 35180295 | rs1071695 | C | T | | exonic | N | N | N | N | N | N | 0,0 |

*CHR: Chromossome; R: Reference Allele; P: Polymorphic allele; %D: Deleterious percentage; D: Deleterious; N: Neutral*

|  | **#CHR** | **POS** | **ID** | **R** | **P** |  | **PredictSNP2.0** | **CADD** | **DANN** | **FATHMM-MLK** | **FUNSEQ2** | **GWAVA** | **%D** |
| --- | --- | --- | --- | --- | --- | --- | --- | --- | --- | --- | --- | --- | --- |
| RASSF5 | 1 | 206513930 | rs12124627 | C | G | intronic | D | D | D | D | ? | D | 83,3 |
|  | 1 | 206539214 | rs7530746 | A | T | intronic | D | D | D | D | D | N | 83,3 |
|  | 1 | 206555654 | rs72755019 | C | T | intronic | D | D | D | ? | D | D | 83,3 |
|  | 1 | 206557363 | rs67223538 | G | A | UTR5 | D | D | D | ? | D | D | 83,3 |
|  | 1 | 206584525 | rs4845112 | C | T | exonic | D | D | D | N | D | D | 83,3 |
|  | 1 | 206512396 | rs871782 | C | T | intronic | D | D | N | D | D | N | 66,7 |
|  | 1 | 206539214 | rs7530746 | A | G | intronic | D | D | N | D | D | N | 66,7 |
|  | 1 | 206557313 | rs71633591 | C | A | UTR5 | N | D | D | N | D | D | 66,7 |
|  | 1 | 206559319 | rs11119043 | G | A | intronic | D | D | D | N | D | N | 66,7 |
|  | 1 | 206580154 | rs11119083 | G | A | intronic | D | D | D | ? | D | N | 66,7 |
|  | 1 | 206588606 | rs11589 | T | C | UTR3 | D | D | D | N | ? | D | 66,7 |
|  | 1 | 206506970 | rs3813976 | C | T | upstream | N | D | D | N | D | N | 50,0 |
|  | 1 | 206555654 | rs72755019 | C | G | intronic | D | D | N | ? | ? | D | 50,0 |
|  | 1 | 206557363 | rs67223538 | G | C | UTR5 | N | D | N | N | D | D | 50,0 |
|  | 1 | 206563245 | rs4844540 | G | A | intronic | N | N | D | ? | D | D | 50,0 |
|  | 1 | 206563245 | rs4844540 | G | T | intronic | N | N | D | ? | D | D | 50,0 |
|  | 1 | 206573537 | rs7527917 | A | G | intronic | N | D | D | N | D | N | 50,0 |
|  | 1 | 206575854 | rs6660116 | C | T | intronic | D | N | D | ? | D | N | 50,0 |
|  | 1 | 206584525 | rs4845112 | C | A | exonic | N | D | N | N | N | N | 16,7 |
| NTAN1 | 16 | 15038105 | rs1135999 | A | C | exonic | N | D | D | N | D | ? | 50,0 |
|  | 16 | 15054789 | rs11075253 | C | A | intronic | N | D | D | N | D | N | 50,0 |
|  | 16 | 15057504 | rs2280017 | C | T | intronic | N | D | D | N | D | N | 50,0 |
|  | 16 | 15038105 | rs1135999 | A | C | exonic | N | D | D | N | D | ? | 50,0 |
|  | 16 | 15038105 | rs1135999 | A | G | exonic | N | D | N | N | D | ? | 33,3 |
|  | 16 | 15038117 | rs1136001 | G | A | exonic | N | N | N | N | N | ? | 0,0 |
|  | 16 | 15038117 | rs1136001 | G | C | exonic | N | N | N | N | N | ? | 0,0 |
|  | 16 | 15038117 | rs1136001 | G | T | exonic | N | N | N | N | N | ? | 0,0 |

*CHR: Chromossome; R: Reference Allele; P: Polymorphic allele; %D: Deleterious percentage; D: Deleterious; N: Neutral*
